# Fang Filling

**Published:** 1863-09

**Authors:** W. H. Shadoan


### FANG FILLING.

EDS. REGISTER.—It is not my intention to enter into anything like a discussion of this subject, but to give a few observations on cases reported some time since. In the April number of the *Register* for 1861, I reported a few cases which I now wish to examine. In March last (1863), I saw Case 2, operated upon in April, 1859; I find the tooth in excellent condition. The tooth is as serviceable as its neighbor. Has had no difficulty since treatment.

Case 4. Mrs. G. had slight pain for ten minutes once since the case was dismissed. This case was the most aggravated case of alveolar abscess that ever came under my care, and I think it is one of the best cures. The lady avers that she has had the most complete freedom from soreness and pain in that tooth of any she ever had filled.

*Case* — Mr. Allen, (this case has never been reported), a gentleman of 68 years of age, had disease of nerve of 2nd Sup. left bicuspid, which I treated on the 1st Dec., 1859. Treated it as others, using metallic arsenic, and creosote to destroy the nerve, and then creosote alone. I examined the tooth on the 5th of August, 1863. He says the tooth is as free from pain and soreness as though it had never been diseased.

In the July number of the *Register*, 1862, I said that I had used for filling the crown cavity of a tooth, for a gentleman, after treating as described, Roberts' Os-Artificial, and gave my reasons for it. The tooth was very frail. A short time since he took a small stick between his teeth, and accidentally broke the crown off. On examination the gold in the fang, was forced only opposite the border of the alveolar process.

I had saturated a small pledget of cotton with creosote and forced it up into the fang as far as I could conveniently, then packed the gold up against the cotton, with an instrument made for the purpose. (Not a broach). When the tooth broke off, the gold was found well packed. I examined the bone of the tooth to ascertain what effect the crown filling had had upon the tooth, and I could see no difference in the Os-Artificial filling and that of gold. The tooth was dry and hard. I have in my mind, another case of a similar character. It had given the patient, a gentleman of 45 years of age, considerable pain and trouble for five years; I treated it in the usual way, and filled as in the preceeding case. The tooth is to all appearances as useful as ever before. He has no trouble with it whatever. One difference in that tooth, and others filled with gold entire is, that it never is affected with cold and heat as they are.

I am not an "enthusiast," but I have great faith in the careful management of diseased fangs of teeth. I have for seven years been treating fangs of teeth whenever I thought the case would at all justify, and in a few instances I have undertaken cases when I had but little hope of curing. I

think from what I have seen of this class of work, that I have cured at least nineteen in twenty. If, instead, we would lose nineteen in twenty, would it not be better than to loose all in not treating any of them.

W. H. SHADOAN.

